# Laryngology and Otology

**Published:** 1909-03

**Authors:** P. Watson Williams


### LARYNGOLOGY AND OTOLOGY.

**Abnormal Pulsating Vessels in the Pharynx.**—It is hardly necessary to direct attention to the importance of noting the existence of large and abnormal pulsating vessels in the pharynx, prior to operation on the implicated region, and perhaps especially in view of the frequency with which operations for the removal of adenoids are undertaken. These abnormalities, fortunately, are not common; but in this lies the danger, as their presence is but seldom suspected or sought.

Connal[1] recently showed a series of nine at a meeting of the Glasgow Medico-Chirurgical Society, and at the same time read notes of three others that he had observed. In some the pulsating vessel was bilateral. The condition was met with in six women, three men, and three young boys. The pulsating vessel is situated behind the posterior pillars of the fauces, and in most cases ascends to the naso-pharynx. In three cases the vessels were bilateral, and where unilateral the vessel was seven times on the right side, and twice on the left side. In eight cases the pulsation was marked, and gave the impression of a large vessel. In the three boys' cases—aged respectively six, ten and eleven years—nasal obstruction, with enlarged tonsils and adenoids, existed. In one of the boys the pulsation was bilateral and very marked, communicating a heaving impulse to the left tonsil, which was hypertrophied.

Connal reminds us that most observers have ascribed the pulsation to the ascending pharyngeal artery, but that Schmidt and Kelly attribute the abnormal pulsation to the internal carotid artery. Connal believes that these large pulsating vessels are sometimes the ascending pharyngeal, and sometimes a tortuous internal carotid artery. He further points out that there is sometimes a slight difficulty in detecting them. The movements of the posterior faucial pillars, and of the lateral walls of the pharynx, are apt to mask the pulsation, while in the slighter cases the movements of the tongue must be well under control.

1 *J. Laryngol., Rhin. and Otol.*, 1908, xxiii, 130.

**Bárány's Tests** have been employed by Pike[1] in "an examination into the condition of the vestibular apparatus in a series of cases of deafness, of non-suppurative origin." The vestibular apparatus includes the membranous parts of the semicircular canals, with the utriculus and sacculus, and the contained endolymph. The semicircular canals are three on each side, viz. the horizontal, anterior and posterior, and the two latter lie at an angle of 45° with the sagittal antero-posterior diameter of the head; hence it follows that the plane of the anterior on one side coincides with the plane of the posterior on the opposite side, and vice versa. Now movement of the head causes movement of the endolymph in one or more of these semicircular canals, and the movement of the endolymph involves movement of the cupola, and thus stimulation of the special hair-cells of the eighth nerve, and as whenever the cupola is moved vestibular nystagmus is produced, provided the rest of the reflex path is intact. It is mainly by observing the nystagmus, after employing certain clinical methods, that we judge of the condition of the vestibular apparatus. The clinical methods employed have been (1) the turning reaction, *i.e.* observation of the nystagmus, produced after turning the patient on a revolving table ten times, and suddenly stopping; and (2) the caloric reaction, *i.e.* the observation of the nystagmus produced by syringing the affected ear with cool water.

*The turning reaction.*—If the patient sits erect during the turning, only the horizontal canals are affected. Having put on a pair of opaque spectacles, he is turned first to the right as the hands of a clock go, and the duration of the nystagmus produced to the left noted. The result is noted thus: 10 × R=25″ would mean that turning to the right ten times produced nystagmus to the left lasting twenty-five seconds. He is then turned to the left in the same manner, and the result noted.

By bending his head 90° either forward or backward or to the side as he sits in the revolving chair, the vertical canals, instead of the horizontal, will be in the horizontal plane, and will be affected on turning; and it is a law that each canal produces an eye movement in its own plane.

One infers that the vestibular irritability is normal when the nystagmus produced by turning to right and left has a duration of twenty-five seconds or over.

*The caloric reaction.*—This can be produced with either hot or cold water, or by cold air or ether vapour. To explain this reaction we must consider that the labyrinth is filled with a fluid at the body temperature, and cold water, for instance, acting through the bony wall will cool this fluid, and a current will be produced, as the fluid cooled will sink to the bottom, and hence the cupola will be moved. Pike states that he has only used cold

[1] *J. Laryngol., Rhin. and Otol.*, 1908, xxiii, 596.

water in the cases he tabulates, an attic cannula being passed into the meatus, and connected by a rubber tube with an ordinary Politzer balloon filled with cold water, so that the ear can be syringed and the eyes watched. It is best to make the patient gaze at you steadily, and then, on pressing the balloon, the nystagmus will be observed. The direction is not constant, but is most often towards the ill side.

Tweedie,[1] who has made "observations on the results of the application of Bárány's tests to deaf mutes," has also employed the **electric test,** *i.e.* the application of the anode and cathode immediately in front of the tragus of each ear, with a continuous current of from twenty to thirty volts, and one to two milli-ampères.

P. WATSON WILLIAMS.

### Reviews of Books.

[1] *J. Laryngol., Rhin. and Otol.*, 1908, xxiii, 592.

